# The biochemical and histological analysis of subcutaneous calcitonin and intramedullary methylprednisolone on bone repair after bone marrow ablation: an experimental comparative study in rats

**DOI:** 10.1186/s40634-017-0100-x

**Published:** 2017-07-20

**Authors:** Salim Ersozlu, Bartu Sarisozen, Ozgur Ozer, Saduman Balaban Adim, Orcun Sahin

**Affiliations:** 1 Faculty of Medicine, Department of Orthopaedics and Traumatology, Istinye University, Liv Hospital, İstanbul, Turkey; 2 Faculty of Medicine, Department of Orthopaedics and Traumatology, Uludag University, Bursa, Turkey; 3 Faculty of Medicine, Department of Pathology, Uludag University, Bursa, Turkey; 4 Faculty of Medicine, Department of Orthopaedics and Traumatology, Baskent University, Ankara, Turkey

**Keywords:** Fracture healing, Marrow ablation, Calcitonin, Methylprednisolone, Bone repair

## Abstract

**Background:**

Although, glucocorticoid (GC) and calcitonin-induced changes in bone repair have been studied previously, the exact effects of these on fracture healing remain controversial. Hence, the purpose of this experimental study is to determine biochemical and histological effects of locally administrated GC and systemically administrated calcitonin on the kinetics of healing response after bone marrow ablation in rats.

**Methods:**

After having undergone marrow ablation, a steroid-treated group of rats (*n* = 24) received a single dose of intramedullary methylprednisolone (2 mg/kg), a calcitonin-treated group (*n* = 24) received intermittently administrated subcutaneous salmon calcitonin (16 IU/kg), and a control group (*n* = 24) received intramedullary saline (25 μl).

**Results:**

Blood samples taken on days 1, 3, 7, 9, and 15 after ablation showed an increase in serum calcium, alkaline phosphatase (ALP), and phosphate levels in the *Calcitonin* and *Control* groups. Levels of calcium and ALP peaked on day 7 after ablation. However, an increase in phosphate levels indicated a biphasic reaction that peaked on the third and ninth day after ablation. Hypercalcemia was not observed in *Steroid* group because of the inhibition of osteoclastic bone resorption. In that group, the serum levels of ALP and phosphate were lower than baseline levels. The levels of urinary calcium excretion peaked 3 to 7 days after marrow ablation in the control group and 7 to 9 days after that procedure in the steroid group. Histologic evaluation showed that the rats in the control group demonstrated the expected healing period according to the histological grades and that a delay in healing occurred in the calcitonin group after day 9 because of the inhibition of osteoclastic bone resorption. All rats in the steroid group exhibited a decrease and delayed healing response.

**Conclusion:**

Total serum calcium, phosphate, and ALP levels increased after bilateral tibial bone marrow ablation and urine calcium and hydroxyproline excretion also increased as a factor of bone resorption. Subcutaneously administrated salmon calcitonin did not affect biochemical changes after marrow ablation. Single-dose intramedullary methylprednisolone inhibited extra-tibial bone resorption induced by cytokines after bone marrow ablation.

## Background

Mechanical bone marrow ablation (BMX) is a convenient model of intramembranous bone regeneration, in which the intramedullary bone formation occurring on postoperative days 1 to 8 is followed by the resorption of that bone and the re-establishment of red marrow tissue on postoperative days 9 to 15 (Gazit et al. 1989; Gazit et al. 1990; Liang et al. 1992; Magnuson et al. 1997; Schwartz et al. 1989; Suva et al. 1993). BMX, even that which occurs in 1 limb, promotes osteogenesis and chondrogenesis in distant skeletal sites without a respective stimulation of hard tissue resorption (Muhlrad et al. 1981; Gazit et al. 1990).

Although, glucocorticoid (GC) and calcitonin-induced changes in bone metabolism have been studies previously in the literature, the exact effects of these on fracture healing and resorption remain controversial due to different methodologies, heterogenous study groups, different outcome measures and non-comparative conclusions. It is very well-known in the literature that long-term treatment with GCs leads to osteoporosis caused by the inhibition of bone formation and the stimulation of bone resorption (Hardy et al. 1993; He et al. 2016; Hellewell et al. 1974; Kang et al. 2016; Shi et al. 2015; Wang et al. 2002). The change in bone formation may be a direct effect of steroid treatment, but the increased osteolysis probably results from secondary hyperparathyroidism that is caused in turn by decreased intestinal calcium absorption and increased urinary calcium excretion (Hardy et al. 1993; Suzuki et al. 1983; Yan et al. 2012; Wang et al. 2002). Calcitonin, has also been evaluated in the literature in a wide array and approved for the prevention and treatment of osteoporosis (Li et al. 1996). Results from clinical trials and animal studies have shown that calcitonin inhibits bone turnover and prevents bone loss in early postmenopausal woman Gennari et al. 1992; Reginster et al. 1994; Rizolli et al. 2015), and in ovariectomized rats (Bonucci et al. 1995; Li et al. 1996; Wei et al. 2015; de Vernejoul et al., 1990). It has also been shown to modestly increase bone mass in patients with established osteoporosis (Bonucci et al. 1995; Gennari et al. 1992; Reginster et al. 1994; de Vernejoul et al. 1990). In the literature, calcitonin is most widely applied intermittently by subcutaneous injection, nasal spray, rectal suppository, or vaginal deposition. However, the effects of the continuous administration of calcitonin have received little attention to date (Li et al. 1996). To our knowledge, this is one of first studies in the literature that analyzes the biochemical and histologic effects of both intramedullary GC and subcutaneous continuous calcitonin application on bone repair process in a comparative animal model of BMX.

Hence, the purpose of this experimental study is to determine the biochemical and histologic effects of locally administrated GC and systemically administrated calcitonin on the kinetics of bone healing process via quantitative biochemical blood markers and microscopic analysis and to compare the results in order to determine the exact effects of these hormones.

## Methods

### Study groups

Seventy-two male Sprague–Dawley rats (weight range, 250–290 g) were used for the experimental animal model. The animals were treated ethically in compliance with regulations of Uludag University School of Medicine, Bursa, Turkey, whose quite for the use of experimental animals is based on the principles of laboratory animal care from NIH.

Three study groups (24 rats apiece) were created after right tibial BMX was applied to all 72 rats:

1. Rats with intramedullary GC application (*Steroid group*)
2. Rats with subcutaneous continuous salmo calcitonin application (*Calcitonin group*)
3. Rats with subcutaneous continuous sterile saline application (*Control group*)

Five rats from each group were randomly selected at first, third seventh and ninth day after surgeries (a total of 20 rats for each group) and all were killed with an overdose of sodium pentobarbital. The remaining 4 rats at each group were killed in the same manner 15 days after surgery. The blood samples were obtained by heart puncture. Immediately after the rats had been killed, the right tibia from each animal was placed in cold 70% ethanol and processed for histological evaluation.

### BMX and surgical procedures

All rats were anesthetized by the intraperitoneal administration of ketamine hydrochloride (80 mg/kg) and of xylazine (12 mg/kg). BMX was performed on both tibias of each rat according to the method described by Suva et al. After the proximal region of each tibia had been exposed, a 1.5 to 2.0-mm hole was drilled in the cortex below the proximal metaphysis, and the marrow was removed via several rounds of suction and flushing with sterile saline.

Twenty-five micro liters (μl) of methylprednisolone sodium succinate (2 mg/kg) in sterile saline solution was injected into the intramedullary cavity in the steroid group immediately before wound closure.

Subcutaneous salmon calcitonin application (16 IU/kg) was begun at the first postoperative day and continuously applied per day till the day of sacrifization for each rat in *Calcitonin* group (Li et al. 1996). Similarly, 25 μl of sterile saline solution was applied to the rats in the *Control* group till the day of sacrifization. After the procedures, each rat was housed alone in a standard cage, and food and water access was held unrestricted.

### Blood and urine sample analysis

Blood samples were collected and stored at −40 °C until use. The levels of serum calcium, alkaline phosphatase (ALP), and phosphate were measured in each blood sample. Calcium and phosphorous concentrations were determined by cresolphthalein complexone method and the phosphomolybdate method, respectively. Alkaline phosphatase activity was determined using *p*-nitrophenol phosphate as substrate. An automated analyzer (Parallel, American Monitor Corp., Indianapolis, IN) was used for all these assays. Four rats from each experimental group were maintained individually in metabolic cages for 15 days after surgery, and daily urine specimens were frozen for the urine sample analysis. The first urine sample which was collected during the 24 h-period before BMX was referred as day zero. Levels of urine calcium, creatinin, and deoxypyridinoline (Dpd) were analyzed with an immunoassay method which specifically marks monoclonal anti-Dpd antibody-anticore (IMMULITE Pyrilinks-D, EURO-DPC Ltd., Glyn Rhonwy Llanberis, Gwynedd, United Kingdom).

### Histologic analysis

Immediately after the animals were killed, the samples from right tibias were fixed for 2 to 3 days in 10% neutral buffered formalin, followed by a 2-day period of immersion in Bouin’s solution and were then decalcified in 10% acetic acid, 0.85% NaCl, and 10% formalin. The specimens were then embedded in paraffin, sectioned longitudinally, and stained with hematoxylin and eosin to assess morphological progression of tissue regeneration within the bone marrow cavity. The progression of marrow healing in each specimen was quantified by means of a scale that assigns a grade according to the relative percentage of necrosis, osteoblast, osteoclast, woven bone, and mature bone (Suva et al. 1993). The cell type identification was performed by the morphological features of the specific cell types stained with hematoxylin and eosin under light microscopy. In this 5-step grading system, Grade 1through 5 indicates hemorrhage and necrosis (postsurgical day 1); mesenchymal cell proliferation (day 3); an increase in the number of osteoblasts and the first appearance of woven bone (day 7); a decrease in the number of osteoblasts and transformation to woven bone (day 9); and the presence of both woven bone and mature bone (day 15), respectively.

### Statistical analysis

Comparison data from all samples were obtained via one-way analysis of variance (ANOVA). All tests were controlled with Bonferroni correction. Statistical differences were defined at a confidence level of 95%. The statistical values were given as the mean ± SD. SPSS (Statistical Package for the Social Sciences, version 10.0; SPSS, Inc., Chicago, III, USA) software supported the statistical evaluation. Statistical significance was determined as *p*<. 05 (two-tailed).

## Results

### Blood sample analysis

Table 1 summarizes the mean blood concentrations of the study groups for calcium, phosphate and ALP.

**Table 1:** Results of serum calcium, phosphate and ALP values after BMX

	Days after BMX					
	1 (*n* = 5)	3 (*n* = 5)	7 (*n* = 5)	9 (*n* = 5)	15 (*n* = 4)	*p (ANOVA)*
Blood Calcium level (mg/dl)						
Control group	9.65 ± 1.22	9.47 ± 2.35	12.43 ± 1.69^**a**^	9.85 ± 1.88	9.66 ± 1.89	NS
Calcitonin group	9.23 ± 1.47	9.80 ± 1.61	11.29 ± 0.93 ^**a**^	9.75 ± 1.17	9.37 ± 1.22	NS
Steroid group	9.59 ± 1.33	9.33 ± 0.46	8.96 ± 1.48	9.36 ± 0.71	10.01 ± 2.58	NS
Blood Phosphate level (mg/dl)						
Control group	5.13 ± 1.75	7.96 ± 0.30 ^**b**^	6.39 ± 1.24	10.33 ± 2.89 ^**b**^	4.36 ± 1.52	*.016*
Calcitonin group	4.37 ± 0.97	8.98 ± 1.55 ^**b**^	7.83 ± 1.67	9.39 ± 1.46 ^**a**^	5.30 ± 1.15	*.042*
Steroid group	5.04 ± 1.56	4.74 ± 0.81	4.93 ± 1.64	5.25 ± 0.96	6.65 ± 1.48	NS
Blood ALP level (IU/L)						
Control group	172.3 ±. 11.0	191.7 ± 41.4 ^**a**^	206.7 ± 16.0 ^**a**^	164.5 ± 21.8	154.3 ± 33.0	*.038*
Calcitonin group	128.3 ± 14.7	218.0 ± 86.9 ^**a**^	267.3 ± 29.5 ^**a**^	256.7 ± 45.5 ^**a**^	176.0 ± 71.6	*.004*
Steroid group	157.0 ± 24.7	115.3 ± 27.3	120.8 ± 32.5	133.3 ± 5.6	200.2 ± 64.3	*.009*

All values are expressed as mean ± S.D. ALP: alkaline phosphatase

^**a**^ *p < 0.05,* ^**b**^ *p < 0.01,* significantly different from steroid group

### Calcium

The level of the total serum calcium concentration dramatically increased within 7 days after BMX surgery in the control and calcitonin groups. The average serum calcium values 7 days after surgery for the *Control* and *Calcitonin* groups were 12.43 ± 1.69 mg/dl and 11.29 ± 0.93 mg/dl, respectively. In contrast, no hypercalcemia was detected in the *Steroid* group during the follow-up period of 15 days. In the steroid group, total serum calcium values decreased to a minimum of 8.96 ± 0.48 mg/dl 1 week after surgery. There was statistically significant difference between the *Steroid* group and the other study groups (*p*<. 05 *Steroid* vs. *Control* and *Calcitonin* groups) (Table 1).

### Phosphate

In *Control* and *Calcitonin* groups, the total serum phosphate levels begun to increase on the first day after BMX and reached the maximum level of xxxx on the third and ninth day. The phosphate level for these study groups turned to normal 15 days after surgery. There was no statistically significant difference between *Control* and *Calcitonin* groups (*p*=. 016, *Control* group; *p*=. 042, *Calcitonin* group). The serum phosphate level decreased significantly after BMX in the *Steroid* group compared with the other groups 3 days after surgery (*p*<. 01 *Steroid* vs. *Control* and *Calcitonin* groups) (Table 1).

### Alp

In the *Control* group, total serum ALP levels increased slowly starting from the first day (172.3 ± 11.0 IU/L) after BMX and reached the maximum level of 206.7 ± 16.0 IU/L on the seventh day. In the Calcitonin group, the mean value for ALP was 128.3 ± 14.7 IU/L on the first day after BMX and it increased to 267.3 ± 29.5 IU/L on the seventh day. This increase of ALP for both study groups was found statistically significant (*p*<. 05 for both). In the *Steroid* group, ALP levels decreased on the third and seventh day after BMX surgery (115.3 ± 27.3 IU/L and 120.8 ± 32.5 IU/L, respectively). The ALP levels of the *Steroid* group was found significantly different compared with the Control and Calcitonin groups (*p*<. 05 *Steroid* vs. *Control* and *Calcitonin* groups) (Table 1).

### Urinary analysis

#### Dpd

The level of urinary Dpd in the *Control* group decreased on the first day after BMX and then started to increase from the day 7 and reached the maximum level on the ninth day (*p*<. 01, compared with the levels on days 0, 1, 3, and day 15). Nevertheless, urinary Dpd levels sharply decreased again on the fifteenth day after BMX (Table 2).

**Table 2:** Results of urine calcium, Dpd, and creatinin values after BMX

		Days after BMX					*p* (ANOVA)
	Day 0 (*n* = 8)	1 (*n* = 8)	3 (*n* = 8)	7 (*n* = 8)	9 (*n* = 8)	15 (*n* = 4)	
Urine Calcium level (mg/dl/24 h)							
Control group	6.42 ± 1.73	6.91 ± 1.57	15.61 ± 2.97 ^ce†^	23.74 ± 7.91 ^‡^	8.74 ± 2.91	9.45 ± 2.78	*< .0001*
Calcitonin group	6.42 ± 1.73	6.84 ± 2.44	6.27 ± 1.82	9.14 ± 3.28 ^ae^	8.40 ± 2.14	7.85 ± 1.92	NS
Steroid group	6.42 ± 1.73	6.81 ± 3.58	6.83 ± 3.56	20.08 ± 7.14 ^†^	31.47 ± 6.35 ^bd‡^	19.41 ± 8.42^†^	*< .0001*
Urine Dpd level (μg/day)							
Control group	126.4 ± 28.6	69.2 ± 16.4	117.0 ± 36.5 ^f^	159.6 ± 42.6 ^cf^	227.3 ± 84.4 ^df†^	72.4 ± 16.8	*.0026*
Calcitonin group	126.4 ± 28.6	51.8 ± 13.3	107.6 ± 16.8 ^e^	89.4 ± 24.3 ^f^	91.5 ± 17.9	138.7 ± 14.6 ^be^	*.0081*
Steroid group	126.4 ± 28.6	48.9 ± 9.4	72.9 ± 7.6	32.9 ± 13.5 ^‡^	72.4 ± 26.4	92.5 ± 22.8	*.0013*
Urine Cr eatinin level (mg/day)							
Control group	122.3 ± 12.5	139.3 ± 44.5	102.9 ± 38.4 ^e^	82.3 ± 31.5 ^e^	80.0 ± 45.3	82.6 ± 56.5	*.017*
Calcitonin group	122.3 ± 12.5	143.0 ± 44.1	131.2 ± 55.3	118.2 ± 34.1	132.6 ± 70.8	102.5 ± 27.3	NS
Steroid group	122.3 ± 12.5	141.8 ± 27.9	175.8 ± 45.8	175.1 ± 88.8	94.2 ± 38.4	99.5 ± 22.1	*.005*

All values are expressed as mean ± S.D. Dpd: deoxypyridinoline

^a^ *p < 0.05,* ^b^ *p < 0.01,*significantly different from control group

^c^ *p < 0.05,* ^d^ *p < 0.01,* significantly different from calcitonin group

^e^ *p < 0.05,* ^f^ *p < 0.01,* significantly different from steroid group

^†^ *p < 0.05,* ^‡^ *p < 0.01* significantly different from day 0

The urinary Dpd levels of the *Calcitonin* group were similar to that of the control group, but the maximum urinary excretion occurred on day 15. Dpd levels of the *Calcitonin* group was significantly different compared with the other study groups (*p*<. 05 vs. steroid group, and *p*<. 01, vs. control group).

In the *Steroid* group, the urinary Dpd values slowly increased on the third day and the lowest value was noted on day 7. The urinary Dpd excretion then started to increase from day 9 until the day 15 (Table 2). Dpd levels of the *Steroid* group was significantly different compared with the other study groups (*p*<. 01 for both groups).

### Calcium

Urinary calcium excretion in the *Control* group increased nearly 4-fold over pre-ablation level 7 days after BMX. This increase was not statistically significant (*p*=. 0021, day 7 vs. day zero). In the *Calcitonin* group, the urinary calcium excretion level did not significantly change in this 15-day study period (*p* > .05). Urinary calcium excretion also increased after BMX in the *Steroid* group; however, the maximum increase occurred 9 days after BMX. On contrary to *Control* group, this increase was found statistically significant (*p*=. 004, day 9 vs. day zero). (Table 2).

### Histologic analysis

#### Control group

**Fig. 1:**
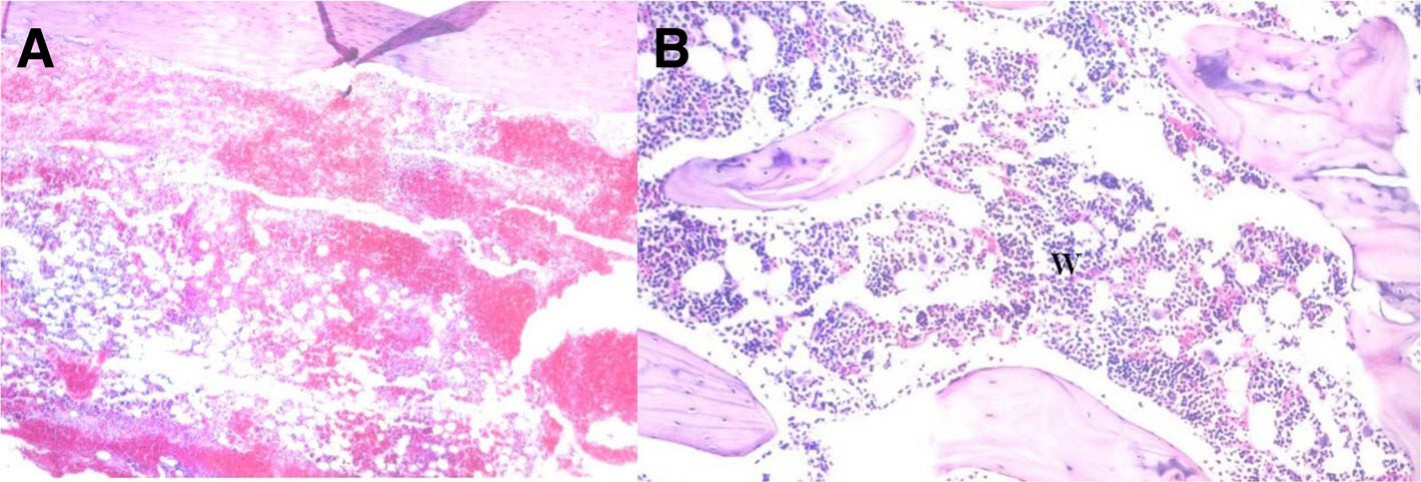
Hematoxylin- and eosin-stained histological sections of *Control* group. **a** A large blood clot was revealed within the within the first hours (X40). **b** After 15 days, the marrow cavity was filled with woven bone and trabecular bone (X200)

The histological sections are randomized and evaluated in a blinded assessment by 2 pathologists specified in musculoskeletal problems. Within the first several hours after ablation in the *Control* group, the formation of a large clot was identified within the marrow cavity (Fig. 1a). Three days after ablation, a cellular structure had formed by a large number of mesenchymal cells. On day 7, the medullar space was densely lined with osteoblasts and small amount of new bone formation was noted at those sites. After that period, most of the surface of the intramembranous space contained osteoclasts, which were resorbing the new cancellous bone. Osteoblasts that covered the newly formed woven bone also appeared and occupied the excavation marrow space on days 9 and 10. On day 15, the marrow cavity in the *Control* group contained woven bone and trabecular bone (Figure 1b). Histologic analysis of *Control* group revealed an expected healing period according to the histological grades.

#### Calcitonin group

Contrary to the all BMX applied specimens with a complete medullary ablation histologically, mature bone and bone marrow were detected in one of the specimens of the *Calcitonin* group on day 3. After that specimen had been reanalyzed, it was concluded that the medullary ablation was only retained as a small focal injury, not enough to accept as a complete BMX. The other areas of bone marrow seemed to be healthy. Because of this inappropriate BMX, that sample was excluded from the study group.

**Fig. 2:**
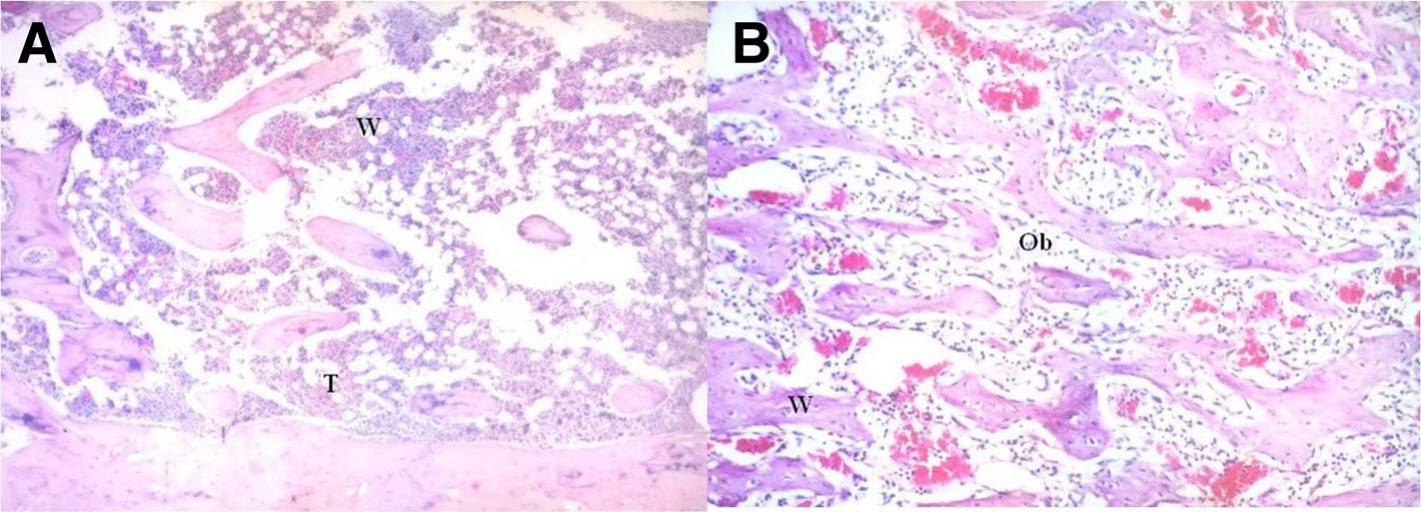
Hematoxylin- and eosin-stained histological sections of *Calcitonin* group. **a** After a week, the healing process was faster than expected. Trabecular bone and bone marrow development (grade 5) were noted instead of massive osteoblasts and new bone formation (grade 3) (X40). **b** There was a delay in healing on day 15, which was detected by massive osteoblasts and woven bone (X100)

On day 7 after ablation, the healing process was found to be faster than anticipated. In those samples, grade 4 healing (highly trabecular bony areas) and grade 5 (trabecular bone and bone marrow development) were noted instead of grade 3 (massive osteoblasts and new bone formation) (Fig. 2a). There was a delay in the healing process in 2 of the 4 samples taken on day 15. In those 2 samples, although trabecular bone and mature bone marrow development (grade 5) was expected, massive osteoblasts and new bone formation (grade 3) was detected (Fig. 2b). The non-developed bone marrow was accepted as a sign of delay in healing for 5 to 6 days.

#### Steroid group

**Fig. 3:**
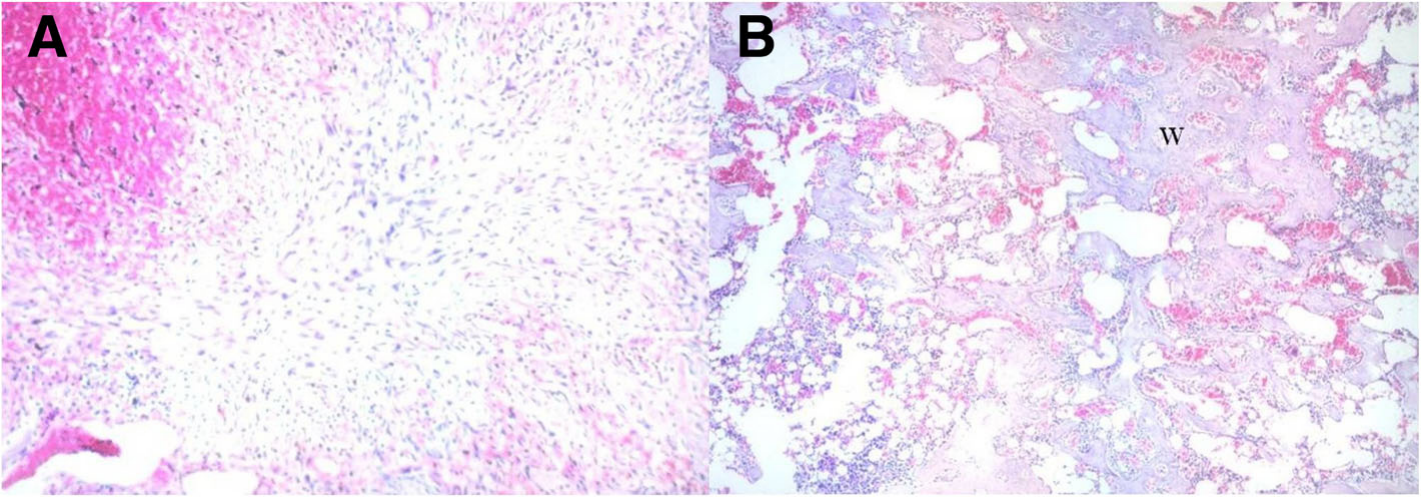
Hematoxylin- and eosin-stained histological sections of *Steroid* group. **a** Tibial healing delayed and the bleeding and necrosis were detected on day 3 (X100). **b** The marrow cavity filled with woven bone instead of trabecular on day 15 (X40). **T**, trabecular bone; **W**, woven bone; **Ob**, osteoblast

Histologic analyses of the *Steroid* group showed that tibial healing was delayed and tissue repair occurred less quickly than expected for all samples. Although significant numbers of mesenchymal cells were expected on the third day after BMX (grade 2 healing), the samples taken on the third day had only bleeding and necrosis (grade 1 healing) revealing a delay in bone repair (Fig. 3a). Similarly, samples taken on day 9 after BMX also showed a delayed bone healing with microscopic features of samples taken on days 5 to 7, which demonstrated large numbers of mesenchymal cells and osteoblasts. In all 4 samples taken on day 15, newly developed trabecular bone (grade 4 healing) was detected (Fig. 3b) instead of mature bone marrow (grade 5 healing), again revealing a delay in bone repair process.

## Discussion

This is one of the first studies in the literature which compares the biochemical and histopathological effects of intramedullary GC and continuous subcutaneous salmon calcitonin application on bone repair after BMX. The main finding of this study is that although subcutaneously administrated salmon calcitonin has no affect on biochemical changes after marrow ablation, single-dose intramedullary methylprednisolone inhibits extra-tibial bone resorption induced by cytokines.

In the literature, there are dozens of studies analyzing the different aspects of bone repair process. In order to understand this process, BMX has been generally accepted as a reliable methodology. Because of its fast healing process, which is independent from the bone cortex, BMXs in rats is widely used as an animal model for intra membranous fracture repair (Magnuson et al. 1997). In rats, the removal of tibial marrow induces primary mineralization in endosteal bone formation, devoid of cartilaginous phase (Chisin et al. 1988; Kuroda et al. 2005). BMX effectively induces the repair-resorption phase of healing (Gazit et al. 1989; Kuroda et al. 2005; Schwartz et al. 1989; Suva et al. 1993), and it is possible to measure the levels of calcium and hydroxyproline in the urine and blood of the rats studied (Gorski et al. 2000; Magnuson et al. 1997). For these reasons, in our study, we have designed an experimental animal model with BMX in rats for the in-depth analysis of bone repair process and used a previously described methodology albeit safe and effective.

In order to analyze the bone repair process after BMX, there have been various mode of application methods, described in the literature for different agents. In these studies, the intramedullary and intramuscular administration of GC and subcutaneous administration of SC have been evaluated. On contrary to these studies, we have continuously applied SC till the day of sacrifization for each rat. We believe that, different mode of administration may have different results. So, in the current study we compared the outcomes of these different administration methods and try to understand the effects for different agents.

### Biochemical changes

**Fig. 4:**
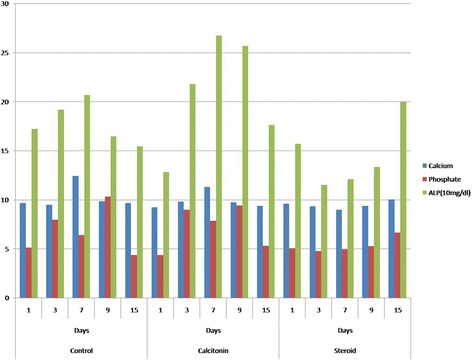
The boxplot graph of *biochemical analysis* for the study groups in regard to specific time intervals

Although some studies in the literature have already investigated the histopathological changes of bone repair, none of them has analyzed the biochemical parameters of the repair-resorption phase and the healing-time relationship (Gorski et al. 2000; Liang et al. 1992; Suva et al. 1993). In a study by Magnuson et al., which analyzed the changes in biochemical parameters that occurred overtime, although the outcomes of GC application were analyzed, the effects of calcitonin were not studied. Magnuson et al. concluded that the total serum calcium level increased after bilateral tibial BMX and blocked with the use of a single dose intramedullary GC administration. Nevertheless, some investigators reported that a decrease in serum calcium levels occurred within 24 h after BMX (Bagnoli et al. 1984; Cooper et al. 1989; Meller et al. 1984). Bagnoli et al. assumed that the decrease in the serum calcium levels was due to a high level of plasma calcitonin and the inhibition of osteoclastic resorption. We have comparable results with the literature in regard to serum calcium levels. In our study, hypercalcemia was detected in the calcitonin group 3 to 7 days after ablation, as it was in the control group (Fig. 4).

Several studies suggest that serum phosphate levels decrease immediately after fracture, increase after 24 h, remain high, and then slowly decrease again, possibly in relation to the period of disability (Meller et al. 1985; Speed 1931). Hardy et al. reported that the decrease in serum phosphate levels immediately after fracture and the increase, later, was related to the serum calcium levels. In our study, we also have found an association between serum phosphate and calcium levels, but the decrease in the serum phosphate levels on day 7 could have been due to the biochemical difference between humans and rats.

ALP activity is often used clinically as a marker of bone formation because of its correlation with calcification. Chondroblasts, osteoblasts and odontoblasts, which are characterized by ALP-specific activity, may control the initial phase of mineralization via matrix vesicles which can be found in various normal and pathologic tissues (Boskey 1981; Schwartz et al. 1989; Wuthier 1982). In a study analyzing the changes of ALP activity during alveolar bone repair in matrix vesicles, Muhlrad et al. reported a decrease in the activity of ALP on the third day of healing and an increase on the ninth day. The changes in ALP levels as a result of bone repair differ according to the study models. In an animal model by Muhlrad et al., granulation tissue formation and new bone formation (characterized by) (characterized by an increase in ALP on the ninth day) were evaluated. It was concluded that although a decrease in ALP on the third day was due to granulation tissue formation, on the ninth day, an increase of ALP was seen because of the new bone formation. In our study, direct endosteal new bone formation was used as a model and contrary to the literature, a decrease of ALP level was detected in the *Steroid* group. The results of *Control* and *Calcitonin* groups had comparable outcomes with the literature.

### Urine analysis

**Fig. 5:**
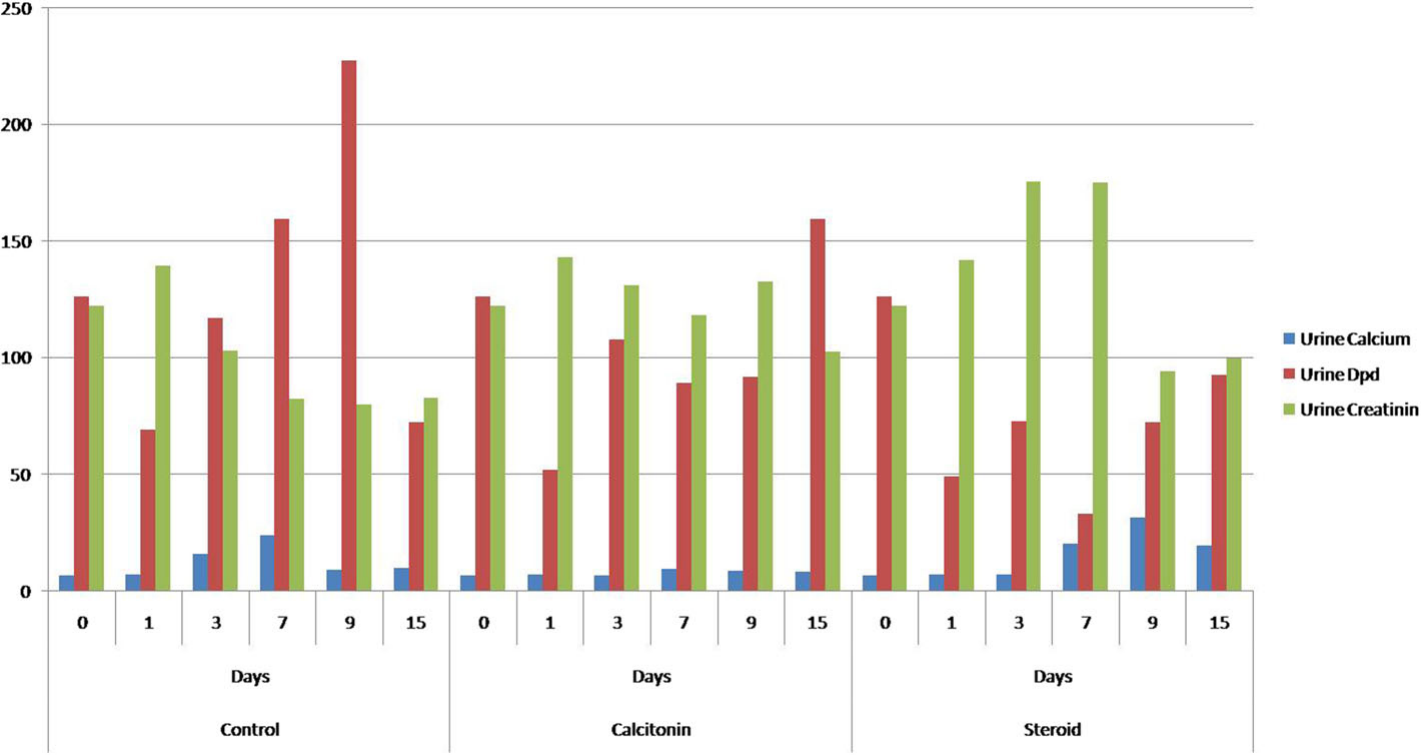
The boxplot graph of *urine analysis* for the study groups in regard to specific time intervals

In the literature, several studies claimed that long-term glucocorticoid usage could have increased calcium excretion in urine as a sign of bone resorption (Hahn et al. 1981; Magnuson et al. 1997). On contrary, Suziki et al. attributed that the reason for increased calcium excretion in urine was due to the decreased reabsorption from the kidneys. In our study, urinary calcium excretion began to increase immediately after BMX and reached its highest level on day 7 in the *Control* group. In the steroid group, urinary calcium excretion began to increase on day 3 after BMX and reached its highest level on day 9 (Fig. 5). It was previously indicated in the literature that the increased urinary calcium levels 3 to 8 days after BMX was a result of 2 mechanisms: resorption from the extra-tibial skeleton and resorption from the newly developed bone tissue in the tibial medullary cavity (Magnuson et al. 1997). In our study, on contrary to the control group, the excretion of calcium was delayed 2 to 3 days in the *Steroid* group, We believe that this delay was caused by methylprednisolone, which initially inhibits cytokines and causes decreased bone resorption in 1 to 7 days before affecting the intestines and the kidneys in 8 to 15 days (Hahn et al. 1981; Reid & Ibbertson 1987; Suzuki et al. 1983).

### Histopathologic evaluation

The literature contains various studies analyzing the effects of different parameters on the repair process of bone. The experimental animal models reveal the different stages of bone repair process and the possible factors that favor or inhibit that process. Medullary bone marrow fills the medullary cavity in a 15-day period in marrow ablation model (Gazit et al. 1990; Magnuson et al. 1997; Suva et al. 1993). In that model of BMX, the healing process is intramembranous ossification and the stages of callus formation (fibrous-cartilage-bone) that is expected in the other healing models, has not been seen in this model (Magnuson et al. 1997; Suva et al. 1993). In our study, a normal healing process was demonstrated by the histologic analysis of samples from the *Control* group. Although the same healing process was observed in the *Calcitonin* group, we detected a delay in the healing process after 9 days. This delay may have been a result of the inhibitory effect of calcitonin on osteoclastic resorption. In the histologic analyses from the *Steroid* group, a decreased or delayed healing response was found in 5 (41.6%) of 12 samples, a result supported by other published studies (Gazit et al. 1989; Gazit et al. 1990; Magnuson et al. 1997; Schwartz et al. 1989; Suva et al. 1993).

## Conclusion

We concluded that in our study, total serum calcium, phosphate, and ALP levels increased after bilateral tibial BMX and urine calcium and hydroxyproline excretion also increased as a factor of bone resorption. Subcutaneously administrated salmon calcitonin did not affect biochemical changes after BMX but has a negative effect on histopathological repair process of bone after 9 days of continuous administration. Single-dose intramedullary methylprednisolone inhibited extra-tibial bone resorption induced by cytokines after BMX. So we believe that, these agents may have a potential to be used clinically in bone fracture repair in the future for those who have a high risk for surgical treatment.
